# Geographic Distribution of the Genus *Panstrongylus* Berg, 1879 in the Neotropic with Emphasis on *Trypanosoma cruzi* Vectors

**DOI:** 10.3390/tropicalmed8050272

**Published:** 2023-05-11

**Authors:** Evelyn Tineo-González, Rossy Fermín, Ana Bonilla-Rivero, Leidi Herrera

**Affiliations:** 1 Biological Diversity Research Laboratory, Natural Sciences Research Centre, Experimental Pedagogical University Libertador, Caracas 1020, Venezuela; evelyntineo@gmail.com (E.T.-G.); rossyfermin8801@gmail.com (R.F.); 2 Post-Graduation Program in Zoology, Faculty of Sciences, Central University of Venezuela, Caracas 1040, Venezuela; 3 Ichthyology Laboratory, Center Museum of Biology UCV, Institute of Zoology and Tropical Ecology, Faculty of Sciences, Central University of Venezuela, Caracas 1040, Venezuela; ana.bonilla@ciens.ucv.ve; 4 Laboratory of Biology of Vectors and Parasites, Center for Ecology and Evolution, Institute of Zoology and Tropical Ecology, Faculty of Sciences, Central University of Venezuela, Caracas 1040, Venezuela; 5 Department of Tropical Medicine, Health Sciences Research Institute, National University of Asunción, San Lorenzo 2160, Paraguay

**Keywords:** geographic distribution, *Panstrongylus*, Neotropic, *Trypanosoma cruzi*, vectors

## Abstract

*Panstrongylus* is a Neotropical taxa of 16 species, some more widespread than others, that act as vectors of *Trypanosoma cruzi*, the etiologic agent of Chagas disease (CD). This group is associated with mammalian reservoir niches. There are few studies of the biogeography and niche suitability of these triatomines. Using zoo-epidemiological occurrence databases, the distribution of *Panstrongylus* was determined based on bioclimatic modelling (DIVA GIS), parsimonious niche distribution (MAXENT), and parsimony analysis of endemic species (PAE). Through 517 records, a wide presence of *P. geniculatus*, *P. rufotuberculatus*, *P. lignarius*, and *P. megistus* was determined and recorded as frequent vectors of *T. cruzi* in rainforest habitats of 24–30 °C. These distributions were modeled with AUC >0.80 and <0.90, as well as with the seasonality of temperature, isothermality, and precipitation as relevant bioclimatic variables. Individual traces for each taxon in *Panstrongylus*—1036 records—showed widely dispersed lines for frequent vectors *P. geniculatus*, *P. lignarius*, *P. rufotuberculatus*, and *P. megistus*. Other occasional vectors showed more restricted dispersal, such as *P. howardi*, *P. humeralis*, *P. lenti*, *P. lutzi*, *P. tupynambai*, *P. noireaiui*, and *P. chinai*. Areas of defined environmental variation, geological change, and trans domain fluid fauna, such as the American Transition Zone and the Pacific Domain of Morrone, had the highest *Panstrongylus* diversity. Pan-biogeographic nodes appear to be areas of the greatest species diversity that act as corridors connecting biotopes and allowing fauna migration. Vicariance events in the geologic history of the continent need to be investigated. The geographical distribution of *Panstrongylus* overlapped with CD cases and *Didelphis marsupialis/Dasypus novemcinctus* presence, two important reservoirs in Central and South America. The information derived from the distribution of *Panstrongylus* provides knowledge for surveillance and vector control programs. It would increase information on the most and less relevant vector species of this zoonotic agent, for monitoring their population behavior.

## 1. Introduction

The genus *Panstrongylus* Berg, 1879, belongs to the tribe Triatomini, subfamily Triatominae (Hemiptera, Reduviidae), and comprises 16 living species and one fossil species described from the Dominican Republic [1,2,3]. This taxa is restricted to the American continent and the other different species are found from Mexico to Argentina, some of which are very widespread [4,5,6].

The geographical distribution of triatomines is a determining factor in the epidemiology of American trypanosomiasis (AT) or Chagas disease (CD).

Species of *Pantrongylus* are considered potential vectors of *Trypanosoma cruzi* (Eukarya, Kinetoplastea, Trypanosomatidae), which is the etiological agent of CD. They are found in rural, urban, and suburban dwellings with wildlife corridors and vector domiciliation [7,8,9,10,11]. As a result, when other vectors have been chemically controlled, many of the *Panstrongylus* species have emerged as successors.

Triatomines can be attracted for hematophagy by several orders of mammals that have been reported to be reservoirs of *T. cruzi*, as well as by some birds that may be naturally infected with this parasite [11,12].

*Panstrongylus* is linked to the niches occupied by armadillos, opossums, bats, rodents, anteaters, macaws, porcupines, small reptiles, toucans and domestic animals such as chickens, dogs, cats, and occasionally domestic pets [5].

Some studies of triatominae have used species distribution modelling (SDM) [6]. One dimension of these studies is the approach to biological history and biogeography that allows us to understand the actual and potential dispersal of triatomine species by using a tracing approach based on ecological niche models and parsimony analysis of endemism (PAE) [13].

Another approach is the study of biodiversity carried out by DIVA software version 7.5. It presents data for a species through maps of its geographical distribution using the BIOCLIM algorithm, identifying areas with a similar climate to where the species is found [14]. This can be associated with the maximum entropy statistical approach to ecological requirements for distribution using Maxent software version 3.4.4. (https://biodiversityinformatics.amnh.org/open_source/maxent/, accessed on 13 April 2021) which allows one to predict the distribution of presence, estimate the expected distribution probability, and find the most uniform maximum entropy [15].

Estimates of the potential distribution of insect vectors have improved the analysis of the relationships between environmental factors, vector ecology and the transmission of vector-borne disease agents [15,16].

The present study proposed a biogeographic analysis of the distribution of *Panstrongylus* in the Neotropics, focusing on vector species based on laboratory and literature records using DIVA GIS, MAXENT, and PAE [13,16]. Some important mammalian groups involved in the *T. cruzi* cycle were considered in this modelling.

Bioclimatic variables and historical/ecological events that would delimit *Panstrongylus* presence and support predictive scenarios of CD eco-pathogenic complex elements were considered.

## 2. Materials and Methods

### 2.1. Study Area

The study area corresponded to the geographic distribution of the genus *Panstrongylus*, which is restricted to the Neotropical taxa, specifically from Mexico to Argentina [4,6,17].

### 2.2. Species Database and Georeferencing

Records of triatomine occurrences from technical reports, museum records, biological laboratory collections and specialized bibliography were used to compile a database for each species. All were geo-referenced using DICES platform (http://www.dices.net, accessed on 21 September 2020) and GADM version 2.8 (http://www.gadm.org, accessed on 12 February 2021). The database was cleansed of duplicate occurrences to eliminate sampling effort bias, enable species distribution modelling and pan-biogeographic analysis, and only species separated by more than 1 km were selected to reduce autocorrelation.

### 2.3. Modelling the Geographical Distribution, Diversity, and Species Richness of Panstrongylus and Sympatric Mammals

In total, 11 species of the genus *Panstrongylus* and 2 mammalian taxa, *Didelphis marsupialis* (Ameridelphia; Didelphimorphia) and *Dasypus novemcinctus* (Xenarthra; Cingulata), known *T. cruzi* hosts, all with more than 10 occurrence records, were considered for distribution modelling using MAXENT (version 3.4.4) [11,18,19,20,21,22,23].

The georeferenced database of each species was transformed into a comma-separated values file (CSV) with .csv extension by converting the original grd format into a MAXENT compatible ASCII format (*.asc). DIVA-GIS 7.0 was used as the transformation platform.

In total, 19 layers of bioclimatic variables from global climate database Worldclim were used (http://www.wordclim.org, accessed on 13 March 2021). They were generated from the interpolation of mean temperature, its maximum and minimum, and monthly precipitation for 50 years, with a spatial resolution of 1 pixel = 0.86 km^2^ in Ecuador [20]. An elevation layer available at the same resolution was also considered.

A first run of 10 models was performed using the MAXENT parameters: 1000 iterations, convergence threshold of 1.0 × 10^−5^, 75% of data sets for model calibration and 25% for evaluation. Spearman correlation tests were performed with PAST software version 4.10 on the climatic data obtained with DIVA GIS to reduce collinearity of the variables. Variables with values of <0.7 were included in the model. 

The Jackknife test for AUC (area under the curve) was used to determine the percentage contribution and best fit of the model. After pilot runs of MAXENT, the most predictive variables were obtained and compared with the most biologically relevant variables according to the distribution/habitat where *Panstrongylus* and mammalian species are likely to reproduce and survive [6,21,22,23,24,25,26,27].

Using the selected variables, MAXENT was run again in 10 replicates to obtain an average model for each species [28]. The sensitivity and specificity of the predictions were obtained from the receiver operating characteristic curve (ROC). The area under the curve (AUC) provided a measure of model performance across different limits, with AUCs close to 1 indicating good fit and those near 0.5 indicating poor fit.

The action script file (.asc) output of the MAXENT model was converted with DIVA GIS and visualized on a QGIS map (QGIS version 3.16.10; threshold cut-off at the 10th percentile) for possible areas of suitability for the presence of the different species. In addition, from the database presence of *Panstrongylus* species, spatial analyses of species diversity/richness (alpha diversity) were performed using DIVA GIS.

### 2.4. Pan-Biogeographical Analysis

The construction of individual traces, based on geodetic distance as a starting point, for 12 out of 15 species of *Panstrongylus*, with three or more occurrence records, was carried out using the pattern analysis, spatial statistics and geographic exegesis program (PASSaGE version 2) from a shapefile constructed with the geographic distribution of each species in DIVA GIS [15]. Individual traces were superimposed on the hierarchical biogeographic regionalization of Morrone [29,30], based on biogeographic homologies for the Neotropical region (Figure S1).

Generalized traces were designed from the coincidence of individual unoriented trajectories in minimal spanning trees (MST) based on Jaccard index, compatible with continental scale analysis by PAST sofware.

Zones of convergence between two or more biotic/geological regions (pan-biogeographic nodes) were determined by constructing a binary matrix (1 = presence; 0 = absence) per locality x taxon and a connectivity matrix from MST, driven by the sum of the row values corresponding to the node value/locality and the average node value. Nodes were defined where individual nodal values exceeded the calculated average [29,30].

Parsimony of endemicity (PAE) and maximal parsimony cladistic analyses were used to determine areas of endemism and the most parsimonious cladograms and their corresponding consistency indices (PAST software), with terminal dichotomies indicating more recent biotic exchange with terminal dichotomies indicative of more recent biotic exchanges [13,30,31].

## 3. Results and Discussion

### 3.1. Geographical Distribution Analysis of Panstrongylus Species as Frequent or Sporadic Vectors of T. cruzi

A total of 517 documents containing geo-referenced records of *Panstrongylus* were selected from Scielo, PubMed, BioOne, Redalyc, e-journals, and laboratory records. The geographical distribution of *Panstrongylus* according to the biogeographical regions of Morrone [31] showed that the majority of its species are found in rainforests, between 24 °C and 30 °C (Table 1).

The MAXENT model for widespread of insects vectors, such as *P. geniculatus*, *P. rufotuberculatus*, *P. lignarius*, and *P. megistus*, showed an AUC of >0.80 (robust AUC values based on test and training data are >0.80 and <0.90) [18], which is similar to restricted species such as *P. chinai.*

In general, the best predictive bioclimatic variables were seasonality of temperature (Bio4; standard deviation*100), isothermality (Bio3), and annual temperature range (Bio7) for more commonly infected species (*P. geniculatus*, *P. rufotuberculatus*, *P. lignarus*, *P. megistus*, and *P. chinai*) and for less frequently infected species (*P. diasi*, *P. guentheri*, *P. howardi*, *P. humeralis*, *P. lenti*, and *P. noireaui*) that coincide to a large extent with the bioclimatic conditions recorded in the literature [1,5,14].

The species recorded as frequently infected with *T. cruzi* did not escape the bias of wider coverage (more samples) because of their epidemiological interest. Below is a more detailed description of the distribution of these vector species.

#### 3.1.1. *Panstrongylus geniculatus*

This species was found to be the most widespread and abundant in the Neotropics (293 records). It occurs in different climatic regions, from 360 to 1750 m above sea level (m.a.s.l), such as tropical dry forests, tropical forests, Amazon forests, anthropogenic savannas (in the central and northern regions of Colombia and Venezuela), xerophytic areas of the “Caatinga”/dry grasslands of the “Cerrado” (Brazil), and the areas of influence of the Chaco Domain (ChD) (corridors in Brazil such as Bahia, Minas Gerais, and Goias).

**Figure 1:**
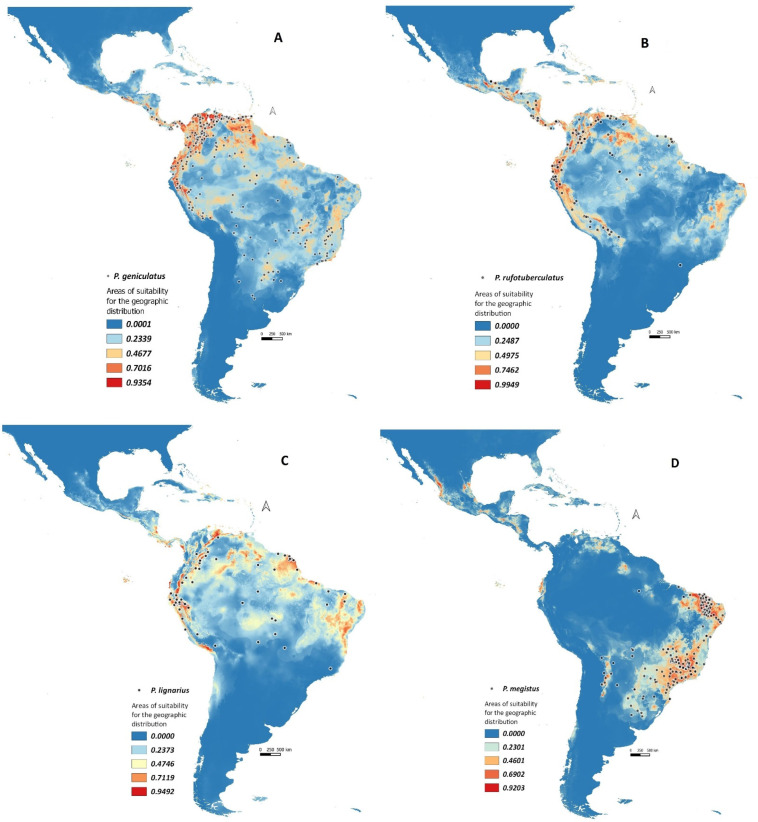
Predictive model of areas of high suitability for geographic distribution of more widespread *Panstrongylus* species, reported as frequent *T. cruzi* vectors in the Neotropics (MAXENT/DIVA GIS). (**A**) *P. geniculatus* in the Mesoamerican, Pacific, and Brazilian Boreal regions (AUC = 0.859). (**B**) *P. rufotuberculatus* in the Pacific and Brazilian Boreal regions (AUC = 0.873). (**C**) *P. lignarus* in Chocó-Darien/Guatuso-Talamanca/Savanna of the Pacific Domain/Roraima and Puna of the Transitional Zone of South America (AUC = 0.866). (**D**) *P. megistus* in the Pacific Domain and Brazilian Boreal (AUC = 0.925).

The potential distribution model (Figure 1A) was generated with a robust predictive value (AUC = 0.859).

Isothermality (Bio3; 24.8%) was the most weighted variable for the model, with the remaining weight equally distributed among the other variables (4.7% on average). This would explain the eclecticism of this species towards different ecoregions, which facilitates the anthropogenic influence on its distribution and reinforces its role as a vector of the trypanosomas [8,10,45,46].

#### 3.1.2. *Panstrongylus rufotuberculatus*

This species (430 records) also had a wide geographical distribution (Figure 1B), covering Central and South America and some Caribbean islands. It was the most widespread after *P. geniculatus* and had a good AUC value (0.873).

The model showed areas of bioclimatic suitability in the province of Pantepui in the Brazilian Boreal Domain (BBD) up to 1700 m.a.s.l., including Rondônia, Acre (Amazon corridors); Mato Grosso (Pantanal) and “Caatinga” (Brazil). Other areas were the Argentine Chaco (349 m.a.s.l) and the northeastern region, closer to the Caribbean coast (between 164 and 1175 m.a.s.l., Venezuela).

The variable that contributed most to the model was the seasonality of temperature (28.1%). Other variables were included in the model, each with a weight of no more than 4%. This would justify the fact that it is an eclectic species in terms of the ecotopes in which it is found and where it could be a potential vector for *T. cruzi* [4,43,47,48,49,50].

#### 3.1.3. *Panstrongylus lignarius*

*P. lignarius* (74 records) had a wide distribution between 177 and 3400 m.a.s.l, including tropical dry forest areas in Brazil (Roraima), Chocó-Darien in Colombia, and Guatuso-Talamanca in Costa Rica, all of which are within the Pacific Savanna domain (PD). The species also occurred in the Ecuador–Perú dyad (Puna; South American Transitional Zone “SATZ”), some Caribbean islands (medium suitability), French Guiana, Suriname, and Venezuela. This would be the first potential distribution model for *P. lignarus* (AUC = 0.866) (Figure 1C).

The bioclimatic variable with the highest contribution to the model was seasonal temperature (Bio 4) (56.8%), another variable with less predictive value was isothermality (Bio 3). Although it is a moderately widespread species, its role as a vector for *T. cruzi* should be studied, given its natural infection and its occasional presence in human dwellings from Brazil and Colombia [51,52].

#### 3.1.4. *Panstrongylus megistus*

*P. megistus* (232 records) showed a wide distribution in humid areas of “Caatinga” and “Cerrado” (230–530 m.a.s.l) Some authors report that the Atlantic Forest is the center of its distribution, with a confluence of populations of this vector in São Paulo linked to rural–urban human migrations, and a great capacity to colonize artificial ecotopes [34,52,53,54].

A suitable bioclimatic area for the occurrence of this species was the MTZ (Sierra Madre West, Sierra Madre East, Sierra Madre del Sur, and Chiapas) and PD (provinces of Western and Guajira), all of which were modeled with a robust AUC (0.925) (Figure 1D).

Seasonality of rainfall (18.8%) was the variable with the highest contribution in the model (low weighting); the other variables would influence the distribution of the species with 4.5% each. This shows that there is no specific bioclimatic condition for its presence, which makes it eclectic and therefore susceptible to anthropogenic effects, some of which are determinants in the epidemiology of CD.

#### 3.1.5. *Panstrongylus chinai*

A distribution in western Ecuador and the Ecuadorian zone of the PD (provinces of Loja and El Oro) was revealed by the occurrence (47 records). In Perú, it was present in the Altiplano ecoregion (east of the province of Puna—3800 or 4000 m.a.s.l), in the Amazon basin of the Pacific slope (0 to 2003 m.a.s.l) and North Andean “Paramo” (NAP).

These distributions were also close to the Colombian province of Cauca at 1560 m.a.s.l (Magdalena), the Savanna of the PD (Napo province of the BBD); Brazilian Southern Domain (BSD; Oyacali and Rondonia) and the Colombian–Venezuelan Guajira (<1000 m.a.s.l), all immersed in variables ecosystems as desert, deciduous forest, dry premontane forest and humid premontane forest.

The variables with the highest contribution to delimiting these suitable areas were isothermality (31.7%) and annual rainfall (30%), indicating precise bioclimatic conditions for its occurrence.

This would be the first possible distribution model for *P. chinai*, which is a potential successor to the primary vector of *T. cruzi.* This was modeled with a good value (AUC = 0.859) and was consistent with bibliographic registers [55,56].

### 3.2. Individual and General Traces of the Distribution of Panstrongylus in the Neotropic

Individual traces for each taxon within the genus *Panstrongylus* showed that some species, frequent vectors of *T. cruzi*, such as *P. geniculatus*, *P. lignarius*, *P. rufotuberculatus*, and *P. megistus* are widely distributed. Other species that are occasionally infected by *T. cruzi* have a restricted distribution, such as *P. chinai*, *P. diasi*, *P. lenti*, *P. lutzi*, *P. guentheri*, *P. howardi*, *P. humeralis*, *P. noireaui*, and *P. tupynambai*.

**Figure 2:**
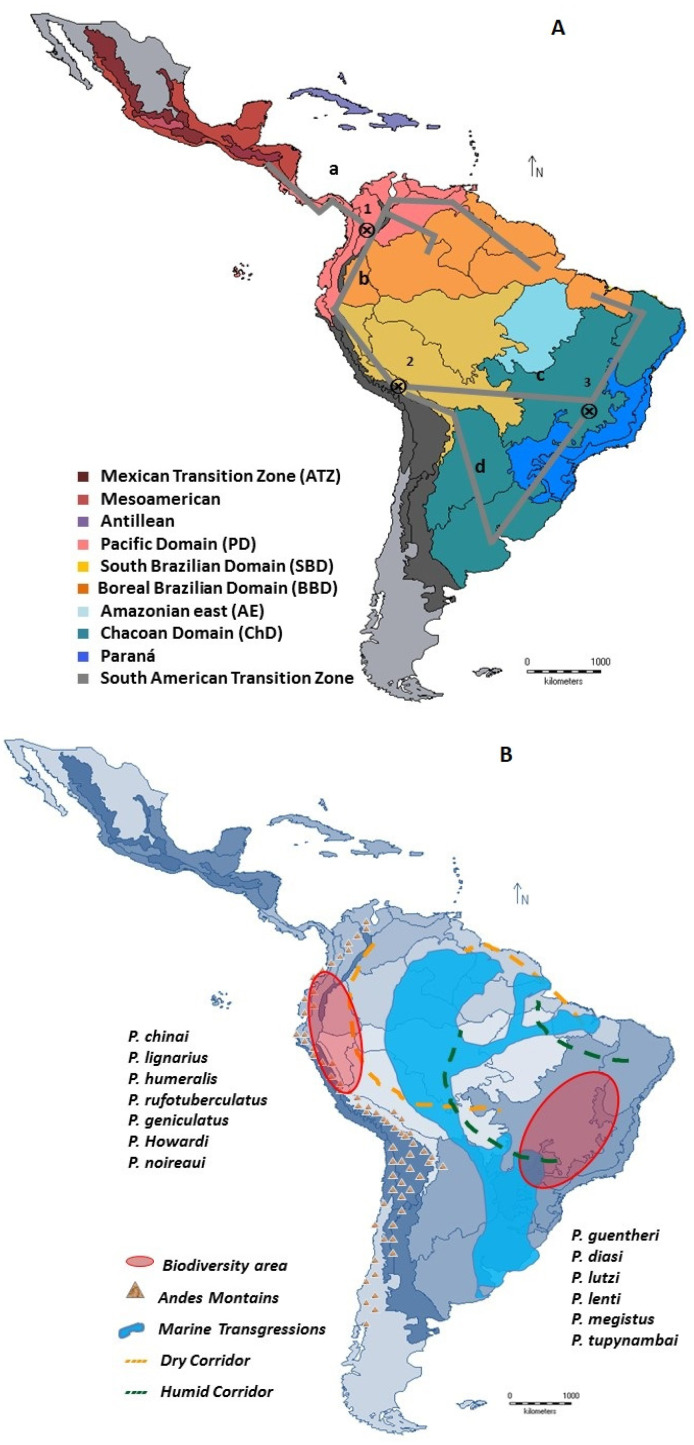
Generalized traces of *Panstrongylus* species and pan-biogeographic nodes (⊗) [26]. (**A**) Schematic of Traces (a–d) and pan-biogeographic nodes (1–3). Trace a: *P. geniculatus, P. lignarius, P. rufotuberculatus*, *P. humeralis*. Trace b: *P. geniculatus*, *P. rufotuberculatus P. lignarius*, *P. chinai, P. howardi, P. martinezorum.* Trace c: *P. geniculatus*, *P. megistus* y *P. rufotuberculatus P. diasi, P. lenti, P. noireaui.* Trace d: *P. geniculatus*, *P. megistus, P. tupynambai*. *P. diasi, P. guentheri, P. lignarius* (⊗) pan-biogeographical nodes (1 “PD”; 2 “SBD”; 3 “Ch D”). (**B**) Patterns of distribution, diversity and endemism of *Panstrongylus* designed on Morrone regionalization.

A generalized trace analysis based on the congruent topology of individual traces for *Panstrongylus* (Table S1; Figures S2 and S3) revealed four traces across the entire Neotropical taxa [57,58] (Figure 2A):

- Generalized trace a. Located in the SATZ (Veracruzana and Chiapas provinces; AMD, Mosquito province) and PD (Guatuso, Puntarenas-Chiriquí, Chocó-Darien, and Magdalena provinces), where *P. geniculatus*, *P. humeralis*, *P. lignarius* and *P. rufotuberculatus* were found.
- Generalized trace b. PD provinces (Venezuelan Guajira and Savanna—east of PD; Cauca, western Ecuador and Ecuadorian zone—west of PD); BBD provinces (Pantepui, Guyana Lowlands); Andean “Paramo” province of SATZ; BD (Napo and Imerí) and BSD (Ucayali, Rondonia, and Yungas) provinces, which include *P. chinai*, *P. geniculatus*, *P. lignarius*, *P. howardi*, *P. martinezorum*, and *P. rufotuberculatus.*
- Generalized trace c. Includes *P. diasi, P. geniculatus*, *P. lignarius, P. lenti, P. lutzi, P. megistus*, *P. rufotuberculatus, P. noireaui* from the BD (Rondônia) through provinces of the Chaco subregion (“Cerrado” and Parana) to areas of the BBD (Para), Chaco domain (ChD) (“Caatinga” and “Cerrado”), and Parana domain (Parana Forest).
- Generalized trace d. Widespread in the Chaco (Chaco and Pampeana departments) and Parana subregions (Parana Forest Department) with *P. diasi*, *P. geniculatus*, *P. guentheri*, *P. lignarius*, *P. megistus*, and *P. tupynambai.*

Triatomini appeared 32 million years ago in the Oligocene, when South American fauna began to migrate towards North America. This coincided with the radiation of mammals (including marsupials and Xenarthra), neotropical birds, and the diversification and distribution of palm species of *Attalea*, *Acrocomia*, and *Butia* throughout South America, which served as micro niches for the triatomines, including specific *T. cruzi* vectors [12,59,60,61,62,63,64].

The distribution of southern cone biota began in the Neogene when the Andes were uplifted and cold ocean currents developed, causing a drier climate towards the Chaco. During the interglacial periods, the fragmentation and mixing of the Yungas and Parana forests with the xerophilous forests of the Chaco may have been a factor in the diversification (vicariance) of the Chaco group (*P. guentheri*, *P. diasi*, *P. lutzi*, *P. lenti*, *P. tupynambai*) [65,66].

The other group was made up of *P. howardi*, *P. chinai*, *P. humeralis*, *P. rufotuberculatus*, *P. lignarius*, *P. geniculatus*, *P. noireaui*, and *P. megistus*, and was associated with the Andean uplift and marine invasions during the Miocene (Pebas Lake), with river channels, swamps, and a characteristic savanna climate [67].

*P. geniculatus*, *P. rufotuberculatus*, *P. lignarius*, *P. howardi*, *P. noireaui* and *P. chinai* converged between the SATZ zone and the PD. In addition, *P. diasi*, *P. lutzi*, *P. lenti*, *P. megistus*, *P. lenti*, *P. rufotuberculatus* and *P. geniculatus* were distributed between the ChD and the Parana domain. Common species in SATZ and PD could be consequence of a trans domain fluid fauna [66]. 

The wide distribution of *P. geniculatus*, *P. rufotuberculatus*, *P. lignarius*, and *P. megistus* could be indicative of their antiquity, which would imply more time for dispersion. The restricted distribution of some species in the PD, the northern region of the SATZ (*P. humeralis*, *P. howardi*, and *P. chinai*), and the ChD (*P. diasi*, *P. guentheri*, *P. lenti*, *P. lutzi*, *P. tupynambai* and *P. noireaui*) would suggest possible speciation events by differentiation and adaptation to specific environmental conditions in each ecotope. These could be recent events (Figure 2A,B).

### 3.3. Species Richness of Panstrongylus, the Associated Mastofauna and the CD Cases in the Neotropical Region

**Figure 3:**
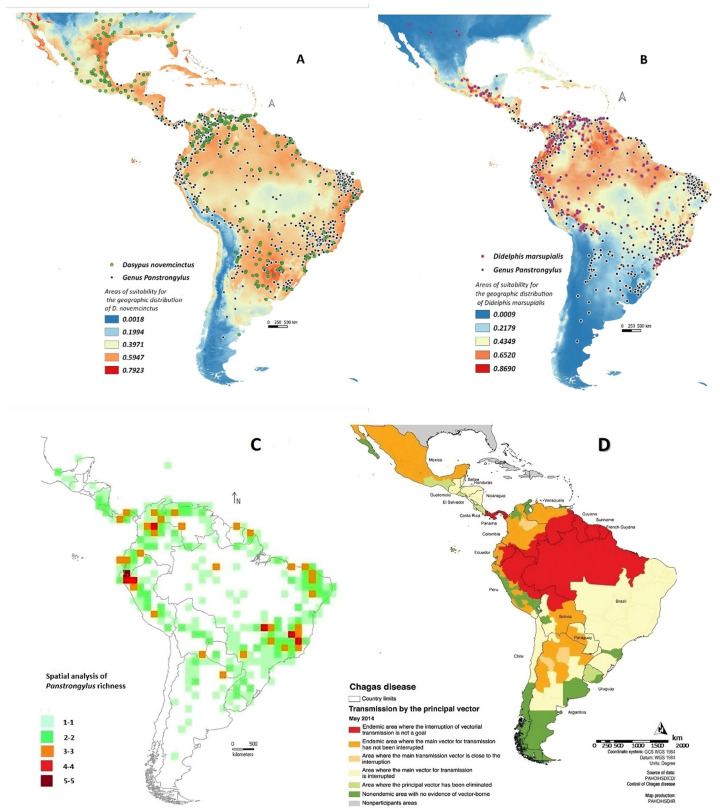
Spatial analysis of *Panstrongylus* richness/associated mastofauna/Chagas disease endemic areas in the neotropical region (MAXENT/DIVAGIS). (**A**) Predictive model of accoupled areas of high suitability for geographic distribution of *Dasypus novemcinctus* (AUC=0.81)/*Panstrongylus* species, (**B**) Predictive model of accoupled areas of high suitability for geographic distribution of *Didelphis marsupialis* (AUC = 0.86)/*Panstrongylus* species. (**C**) Number of *Panstrongylus* species observed/2.5 km of surveyed cells or subunits, with five species/15 of the genus *Panstrongylus* (with the greatest diversity). (**D**) Distribution map of Chagas diseases in Latin America, 2014; modified from PAHO https://n9.cl/g76jf, accessed on 12 November 2021).

The modeled distribution of *D. novemcinctus* (302 records; robust AUC = 0.81) is so wide that it stretches from the southern United States to the middle of Argentina (including some Caribbean islands). It was comparable with *Panstrongylus* distribution in America. Mean annual temperature (Bio1) was the highest contributing variable (28%) in its distribution; annual precipitation (Bio12), mean coldest quarter temperature (Bio11), and temperature seasonality (Bio4) showed 14% (mean) of contribution (Figure 3A).

*Dasypus novemcinctus* would be a versatile reservoir species in terms of the bioclimatic and geomorphological conditions for its presence. It has been reported to be infected with several *T. cruzi* genotypes [11,21,68,69,70].

The distribution of *D. marsupialis* (203 records; robust AUC = 0.86), a primary reservoir in Central and South America (with systemic and anal gland colonization by *T. cruzi*, the latter with infective forms similar to those of the vector) [11,71], was consistent with *Panstrongylus* distribution, restricted to the Neotropics (from Central America to southern Brazil; Figure 3B). Seasonality of temperature (Bio 4) accounted for 56.6% of the distribution, while iso-thermality (Bio 3), mean daily temperature range (Bio 2), and mean temperature of the coldest quarter (Bio 11) accounted for 9% (mean) (Figure 3B).

Spatial analysis of diversity (alpha-diversity) using DIVA GIS showed that the most abundant sites for *Panstrongylus* were located at the junction of provincial boundaries of different areas (15 records of georeferenced *Panstrongylus* species).

Richness analysis revealed two geographical areas with the highest number of *Panstrongylus* species, both coinciding with previously described areas of environmental and geological changes in the Neotropics (generalized traces b, c and part of d, and pan-biogeographic nodes 1 and 2; Figure 2A,B). One of them includes the PD (western Ecuador, Ecuadorian zone and Cauca), SATZ (“Paramo”), SBD (Yungas and Ucayali), and BBD (Napo). The second area was delimited in the CH (Chaco and Parana provinces) with 4–5 species [4,29].

Modelling showed that *Panstrongylus* species richness overlapped with high *Dasypus/Didelphis* (potential blood source for this vector) areas, all coinciding with CD cases (latest 2014). This scenario of overlap would be a potential risk factor for an increase in the incidence of this anthroponotic disease (Figure 3C,D) [11,72,73,74]

The evolutionary history of Xenarthra in the Neotropic is linked to the uplift of the Andes, changes in sea level, temperature variations (transition between the Eocene and Oligocene), and the transition from warm/humid/temperate tropical forest environments to more arid/dry environments dominated by savannas. This explains the distribution of Euphractine in the Chaco and *Dasypus* in the Pacific [60]. These burrowing mammals are considered groups with important zoo geomorphic environmental impacts [69], which should be investigated for eco pathogenic complexes of some zoonoses.

This evolutionary event coincided with the major adaptive radiation of the Didelphimorphia (Ameridelphia, marsupials) in the Late Cretaceous/Early Cenozoic. These taxa diversified when they crossed Australia/America and disappeared from Europe [67,75]. This would explain why some of its species, such as *D. marsupialis*, the primary reservoir of *T. cruzi* in the Americas, are found in a wide range of biomes in the Neotropic [11].

It is also important to note that *D. marsupialis* is the only *T. cruzi* reservoir that has circulating forms of the parasite in its peripheral blood and similar forms of the triatomine infective forms in its anal glands. It therefore acts as both a reservoir and a disperser, ensuring presence and parasite load in the transmission cycle in the absence of a vector [11,71,76].

The highest diversity of *Panstrongylus* was found in rainforest habitats. Seasonality of temperature, isothermality, and precipitation were the most important bioclimatic variables, which is in agreement with the literature.

Species from the Chaco subregion showed wider distributions, with rainfall as a more important variable, whereas temperature was more important for more widespread species from PD.

The geographical distribution of *Panstrongylus* overlapped with CD cases in Central and South America, indicates the need for more monitoring of the most widespread species within these taxa. Individual traces suggest that evolutionary histories would show different trajectories, and that some species may have expanded into atypical regions. One example is *P. chinai*, which is typical of Perú and Ecuador, but may have expanded into the Andean–Venezuelan mountains.

The generalized traces and nodes show that the Andean and Chaco “Cerrado” regions contain the highest number of species of the genus *Panstrongylus*.

The diversification and separation of *Panstrongylus* may have been influenced by the Andean uplift, with the formation of “Caatinga”, “Cerrados”, and marine transgressions, such as the formation of Pebas lakes.

Pan-biogeographic nodes appear to be areas of greatest species diversity that act as corridors connecting biotopes and allowing faunal migration [13]. Vicariance events in the geologic history of the continent need to be investigated in function to explain the origin of the geographic distribution of *Panstrongylus* and the potential impact of its subsequent dispersal.

The same scenario that determined the distribution of *Panstrongylus* led to the diversification of Xenarthra and Didelphimorpha, two mammal taxa associated as blood sources for triatomines.

## 4. Conclusions

This paper presents the first analysis of the geographical distribution of the genus *Panstrongylus* in the Neotropics, focusing on potential vector species of *T. cruzi*, using DIVA GIS, MAXENT and PAE in conjunction. It has also been possible to predict the likely areas of occurrence, which extend the known distribution for almost all of the species.

The modelling of the distribution of the species, with emphasis on the vectors of the CD pathogen, has made it possible to identify areas of greater suitability for the species of the genus *Panstrongylus*.

The vicariance and/or dispersal of such taxonomic groups could be linked to the paleo-ecological processes that took place on the continent.

The models of the species of the Chaco subregion were influenced in a greater importance for the precipitation, while the species of the Pacific domain, with wide distribution, were determined by the variables of temperature.

The geological and paleoclimatic events that led to the diversification of Xenarthra and Didelphidomorpha, mammal groups closely linked to *T. cruzi* transmission cycles, and the diversification of palms, a niche of excellence for triatomines, may be related to the distribution of *Panstongylus.* Further studies will be necessary in the future.

The information derived from *Panstrongylus* models would provide knowledge for the surveillance and vector control programs, as it would increase information on the most relevant species as vectors of zoonotic agents and less studied species for monitoring of their population behavior.

## Figures and Tables

**Table 1:** Distribution and habitat of the *Panstrongylus* species.

Species	Distribution and Habitat	References
* Panstrongylus chinai * (Del Pontei, 1929) *	High jungle in Perú, Ecuador, and localities in the Venezuelan Andes. Found in rocky places and collected in the peridomicile (chicken coops) and inside dwellings.	[4,32,33]
*Panstrongylus diasi* (Pinto y Lent, 1946)	Distributed in “Caatinga” (Brazil), characterized as a semi-arid, warm, and xerophytic area; Santa Cruz (Bolivia), with a warm tropical climate.	[4,17,34]
*Panstrongylus* *geniculatus * (Latreille, 1811) *	From Mexico and Central America to South America (excluding Chile). Found in caves of armadillos, rodents, reptiles (geckos), and bats; also associated with opossums and bird nests, hollow trunks, under the bark of trees, in bromeliads and among palm leaves (*Attalaea*, *Acromia*, and *Copernitia*). Eurythermic species adapted to dry and humid ecotypes such as the Amazon rainforest and xenomorphic grassland areas.	[4,5,6,8,17,35]
*Panstrongylus* *guentheri * (Berg, 1879)	Areas of thorny scrub and dry forest in the Chaco region. Found in armadillo and rodent caves; also associated with opossums. Can be found in the peridomicile associated with woodpiles and environments of goats and dogs.	[4,17,36]
Panstrongylus hispaniolae (†) (Pionar, 2013)	Species are described from a fossil contained in a piece of amber found in the La Toca mine, located in Puerto Plata, Dominican Republic.	[3]
*Panstrongylus* *howardi* (Neiva, 1911)	Equatorial rainforest environment (Manabí province). It has been associated with marsupials and rodent nests inserted in the *Aiphanes eggersi* palm. Found in dwellings and the peridomicile in microhabitats such as piles of bricks or wood coexisting with rodents.	[4,26]
*Panstrongylus* *humeralis* (Usinger, 1939)	Distributed in humid forest from Panama and Colombia.	[4,5]
*Panstrongylus lenti* (Galvão y Palma, 1968)	Habitat typical of “Cerrado”; shrubby, herbaceous, and dry vegetation in Goiás and Bahia (Brazil).	[17,36]
*Panstrongylus lignarius* (Walker, 1837) *	Described from tropical dry forest habitats in Brazil, Colombia, Ecuador, French Guiana, Perú, Suriname, and Venezuela. Found in trees, hollow trunks, and palm trees; it has been associated with toucan nests and mammals such as porcupines and spiny rats. In Perú, it is considered a frequent species in the home and peridomicile.	[17,37]
*Panstrongylus lutzi* (Neiva y Pinto, 1923) *	Endemic to northeastern Brazil (“Caatinga” area). It colonizes armadillo caves and has been found in dwellings associated with rodents and opossums.	[4,17,38,39,40]
*Panstrongylus* *martinezorum* (Ayala, 2009)	Described from a male, collected in Puerto Ayacucho (Amazonas, Venezuela) and other males and females located in collections whose records indicate urban and peri-urban areas of capture.	[2]
*Panstrongylus* *megistus* (Burmeister, 1835) *	Wide distribution in Brazil, with great potential for colonization of artificial environments. In the wild, it is associated with palm trees and animal burrows. The Atlantic forest seems to represent the center of the distribution, although the species is also distributed in humid areas of the “Caatinga” and “Cerrado”.	[17,34,37,41]
*Panstrongylus* *mitarakensis* (Bérenger y Blanchet, 2007)	Distribution in the Mitarakara Mountains, French Guiana.	[42]
*Panstrongylus* *rufotuberculatus* (Champion, 1899).	Most widespread after *P. geniculatus* in Central and South America. Forest habitat, found in palms, hollows and mammal shelters such as bats, monkeys, armadillos. Occasionally in the peridomicile in association with domestic animals.	[4,10,17,37,43]
*Panstrongylus tupynambai* (Lent, 1942)	Atlantic forest region of southern Brazil and Uruguay. Collected in humid rocky habitats, palms, trees, shelters of mammals; human dwellings, and in the peridomicile.	[4,17]
*Panstrongylus noireaui* sp. nov	The specimens were found in the locality of Ayata (Camata), in the department of La Paz, province of Ildefonso de las Muñecas, Bolivia. It is located in the Yunga ecoregion.	[44]

* reported as infected by *T. cruzi* and some vectorial role. † fossil species.

## Data Availability

Not applicable.

## Supplementary Materials

The following supporting information can be downloaded at https://www.mdpi.com/article/10.3390/tropicalmed8050272/s1, Figure S1. Biogeographic regionalization scheme of Morrone of the Neotropical region; Figure S2. Identification of the nodal areas by means of minimum spanning tree with the Euclidean distance index; Figure S3. Identifying nodal areas using Minimum Spanning Tree with Jaccard distance index. Table S1: Area/taxa matrix, with an external area added for reference, used for building minimal spanning trees and parsimony analysis of endemism (PAE).
