# Supplementary figures and images for: Geographic Distribution of the Genus Panstrongylus Berg, 1879 in the Neotropic with Emphasis on Trypanosoma cruzi Vectors
Source: Trop Med Infect Dis. 2023 May 11;8(5):272. doi: 10.3390/tropicalmed8050272 (PMC10223185; doi:10.3390/tropicalmed8050272)

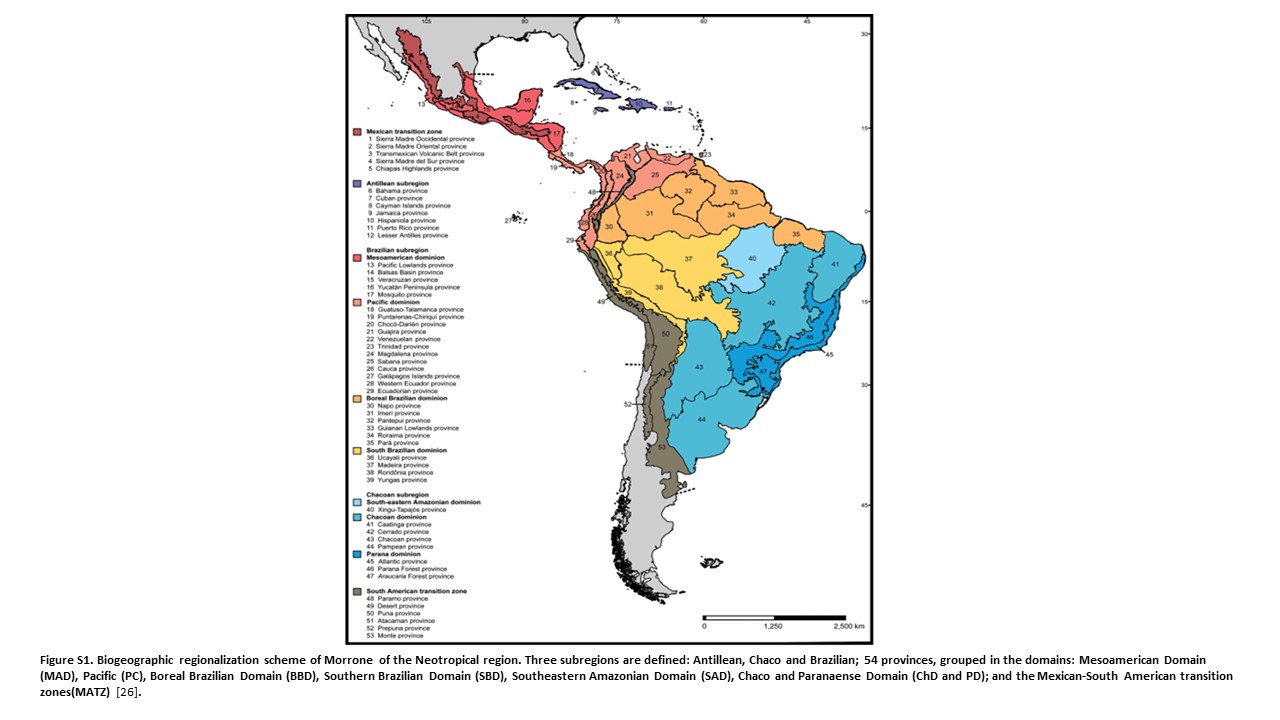

Supplement: Supplementary file 1 [file tropicalmed-08-00272-s001.zip › Figure S1.JPG]

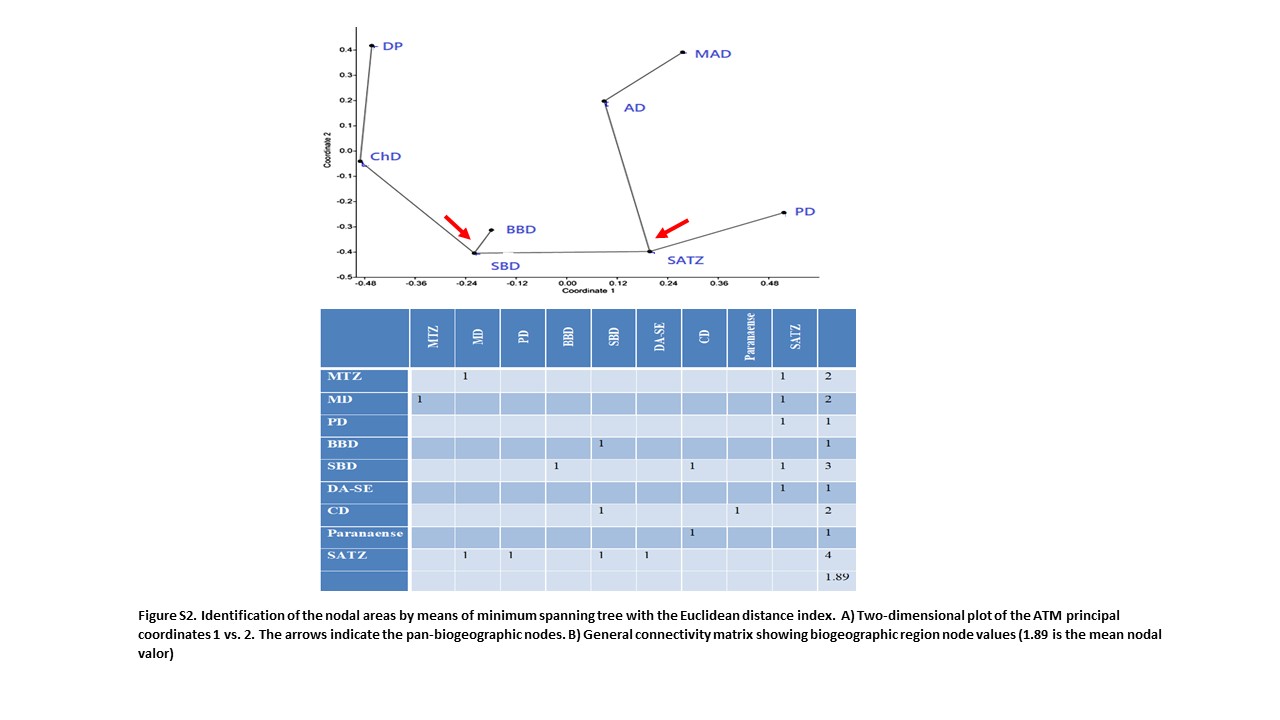

Supplement: Supplementary file 1 [file tropicalmed-08-00272-s001.zip › Figure S2.JPG]

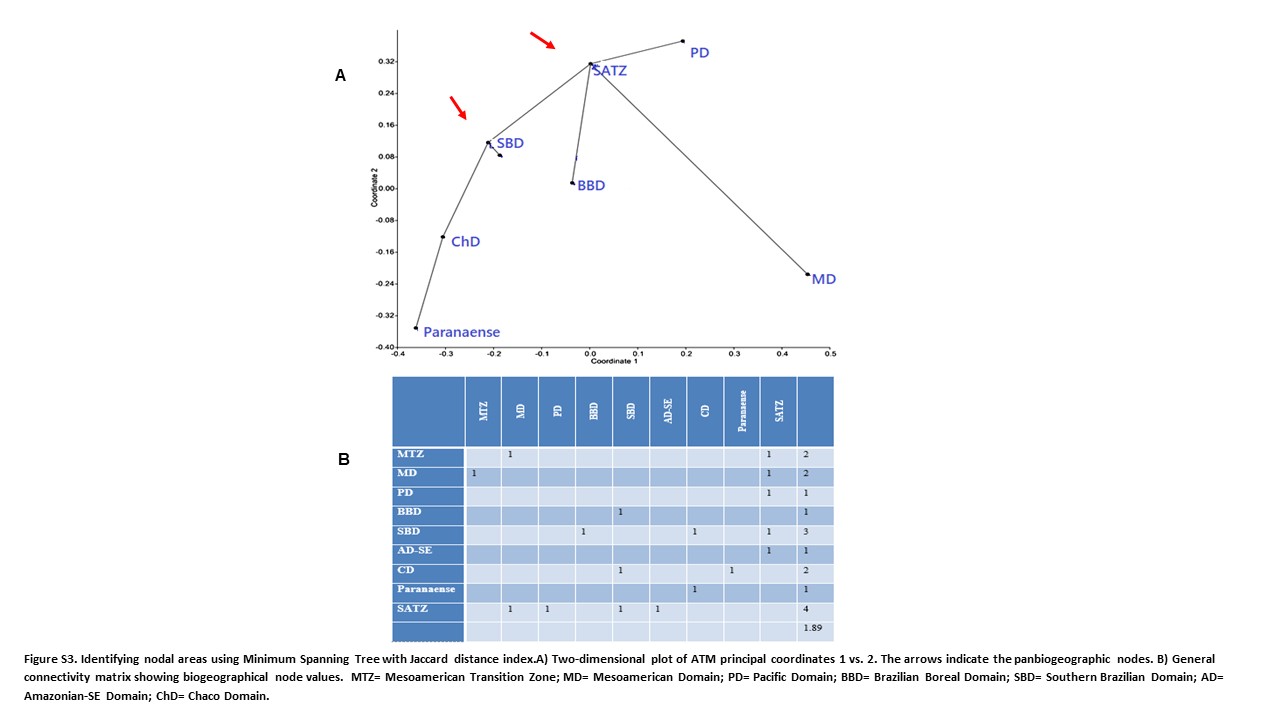

Supplement: Supplementary file 1 [file tropicalmed-08-00272-s001.zip › Figure S3.JPG]

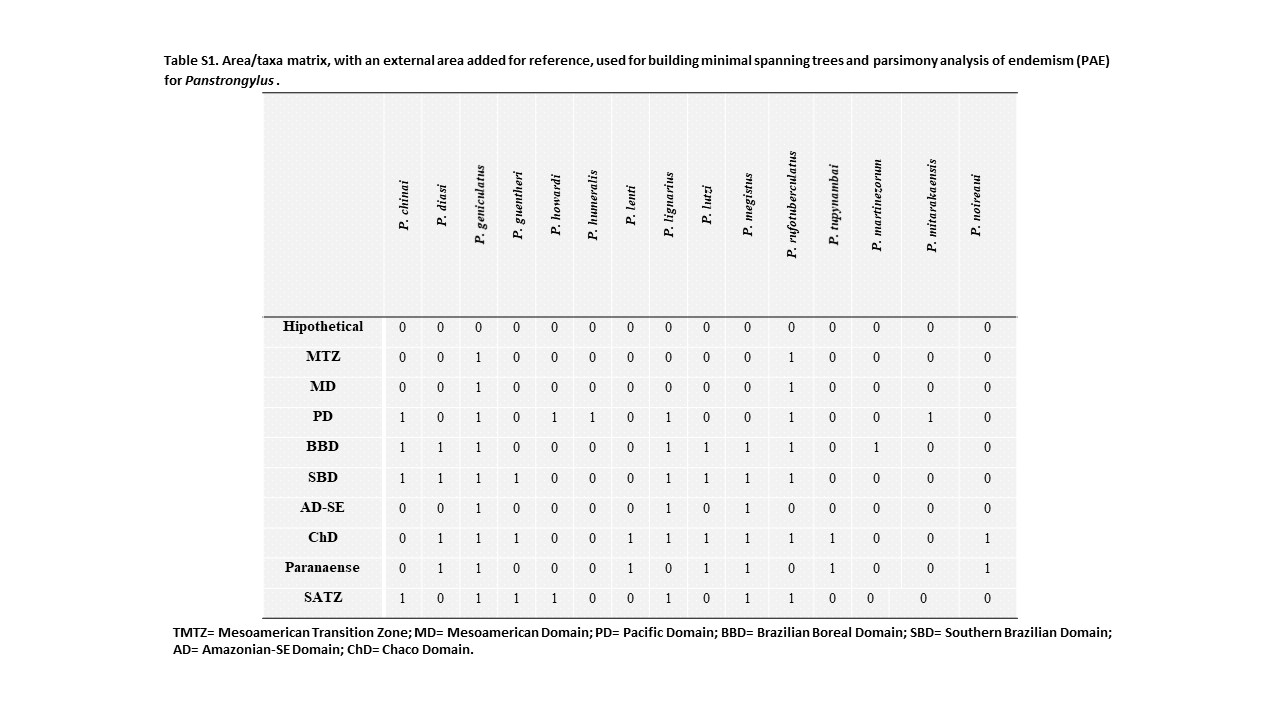

Supplement: Supplementary file 1 [file tropicalmed-08-00272-s001.zip › Table S1 Panstrongylus.JPG]
